# Zearalenone Induces Blood-Testis Barrier Damage through Endoplasmic Reticulum Stress-Mediated Paraptosis of Sertoli Cells in Goats

**DOI:** 10.3390/ijms25010553

**Published:** 2023-12-31

**Authors:** Tengfei Liu, Gengchen Liu, Yinghuan Xu, Yuqi Huang, Yunxuan Zhang, Yongjie Wu, Yongping Xu

**Affiliations:** 1 College of Veterinary Medicine, Northwest A&F University, Xianyang 712100, China; liutf@nwafu.edu.cn (T.L.); lgczs1638@163.com (G.L.); x1648594086@163.com (Y.X.); 1348236038@nwafu.edu.com (Y.Z.); 2 College of Life Sciences, Northwest A&F University, Xianyang 712100, China; yuqihuang@nwafu.edu.cn

**Keywords:** zearalenone, blood-testis barrier, paraptosis, endoplasmic reticulum stress, Sertoli cells, goat

## Abstract

Zearalenone (ZEA) is present worldwide as a serious contaminant of food and feed and causes male reproductive toxicity. The implication of paraptosis, which is a nonclassical paradigm of cell death, is unclear in ZEA-induced male reproductive disorders. In this study, the toxic effects of ZEA on the blood-testis barrier (BTB) and the related mechanisms of paraptosis were detected in goats. ZEA exposure, in vivo, caused a significant decrease in spermatozoon quality, the destruction of seminiferous tubules, and damage to the BTB integrity. Furthermore, ZEA exposure to Sertoli cells (SCs) in vitro showed similar dysfunction in structure and barrier function. Importantly, the formation of massive cytoplasmic vacuoles in ZEA-treated SCs corresponded to the highly swollen and dilative endoplasmic reticulum (ER), and paraptosis inhibition significantly alleviated ZEA-induced SC death and vacuolization, which indicated the important contribution of paraptosis in ZEA-induced BTB damage. Meanwhile, the expression of ER stress marker proteins was increased after ZEA treatment but decreased under the inhibition of paraptosis. The vacuole formation and SC death, induced by ZEA, were remarkably blocked by ER stress inhibition. In conclusion, these results facilitate the exploration of the mechanisms of the SC paraptosis involved in ZEA-induced BTB damage in goats.

## 1. Introduction

Mycotoxin contamination in food and feed has become a major public safety issue around the world, posing serious hazards to human and animal health [1,2]. Zearalenone (ZEA), also known as F-2 toxin, is mainly produced by *Fusarium* fungi and is one of the most widespread mycotoxins that often occur in cereal crops and byproducts [3]. Due to the similarity of its chemical structure to estrogen, ZEA has estrogen-like activity and can competitively bind estrogen receptors, which causes severe reproductive toxicity in humans and animals [4,5]. Numerous studies have documented the primary effects of ZEA on the male reproductive system, and low concentrations of ZEA can cause reproductive damage, such as reducing the levels of testosterone, changing the shape of testes, disrupting spermatogenesis, and decreasing the counts and quality of spermatozoa, ultimately leading to hypo-fertility in male animals [4,6,7]. In male rodents, ZEA treatment elicits strong reproductive toxicity in all ages [4,8,9]. Furthermore, the accumulation of ZEA can impair the quality of male germ cells and decrease the cell survival rate to impede the fertility of farm animals, especially in pigs and poultry [10,11,12]. Similarly, studies in ruminants highlight that dietary exposure to ZEA and its metabolites has adverse effects on male reproductive function, declining male reproductive capacity [11,13,14]. A ZEA threat is now extensively prevalent in the breeding industry and causes significant economic losses; therefore, it is undoubtedly worth elucidating the toxicological mechanism of ZEA-induced disorders of the male reproductive system.

Maturation and development of normal spermatozoa for male fertility require the maintenance of a suitable spermatogenic microenvironment, which depends on the integrity of the blood-testis barrier (BTB) in the testes [15,16]. The BTB is one of the tightest tissue barriers between adjacent Sertoli cells (SCs) within testicular seminiferous tubules and is composed of tight junctions (TJs), gap junctions (GJs), basal ectoplasmic specialization (ES), and desmosomes [16]. The major function of the BTB is to physically divide the seminiferous epithelium into the basal and apical compartments, which can prevent the passage of toxic substances from the blood into the seminiferous tubules and create an immune-privileged environment to protect developing germ cells [15]. Remarkably, many mycotoxin-induced reproductive disorders and spermatogenesis dysfunction have been attributed to the disruption of the BTB integrity in male animals [17,18]. For instance, studies have revealed that deoxynivalenol can induce testosterone deficiency and disrupt the BTB, resulting in the destruction of spermatogenesis in mice [19]. Meanwhile, aflatoxin B1 and T-2 toxins were reported to cause cytotoxicity in SCs and damage to BTB function, leading to impaired spermatogenesis and male infertility [20,21]. Moreover, several previous studies have also demonstrated that ZEA-induced male reproductive toxicity is closely associated with BTB destruction. The reported testicular ultrastructural damage in mice by ZEA exposure was accompanied by decreases in the expression of BTB-related junction proteins and the occurrence of apoptosis or autophagy [22,23,24]. Nevertheless, the processes and mechanisms responsible for ZEA provoking BTB damage and impairing the male reproductive system remain largely unexplored.

Paraptosis is a specific type of programmed cell death (PCD) and morphologically and biochemically differs from apoptosis, lacking a series of apoptotic morphological features, such as nuclear fragmentation, formation of apoptotic bodies, and chromatin condensation [25,26]. The process of paraptosis is morphologically characterized by massive cytoplasmic vacuolization derived from endoplasmic reticulum (ER) dilation and/or mitochondrial swelling [25,27]. As a caspase-independent form of cell death, paraptosis is insensitive to apoptosis inhibition; however, it requires protein synthesis and transcription and can be effectively blocked by the translation inhibitor cycloheximide (CHX) [27]. The ER is a vast membranous network where proteins are synthesized and folded [28]. The accumulation of excessive unfolded or misfolded proteins disturbs the normal physiological state of the ER, triggering ER stress [29,30], which has been implicated in the activation of paraptosis processes [31,32,33]. Moreover, many natural toxins, such as yessotoxin, swainsonine, and honokiol, have been reported to induce paraptotic cell death mediated by ER stress [34,35,36]. Importantly, studies on mycotoxin-induced male reproductive damage have indicated the involvement of diverse types of cell death events, including apoptosis, autophagy, necrosis, and ferroptosis [22,24,37,38]. However, the underlying roles of paraptosis in ZEA-induced mammalian male reproductive system disorders have not been clarified.

In this study, the main aims were to investigate the possible implication of ER stress-mediated paraptosis of SCs in ZEA-induced BTB destruction in goats. The results revealed the adverse effects of ZEA exposure on the BTB integrity, both in vivo and in vitro. Morphological observations and molecular methods validated that paraptotic cell death in SCs was the major reason for ZEA-induced BTB damage in goats. Moreover, ZEA-induced ER stress was closely associated with paraptosis-related vacuole generation. These findings will provide important references for exploring the underlying mechanisms of paraptosis that regulate ZEA-induced BTB damage in goats.

## 2. Results

### 2.1. ZEA Exposure Induced Testicular Dysfunction in Goats

**Figure 1:**
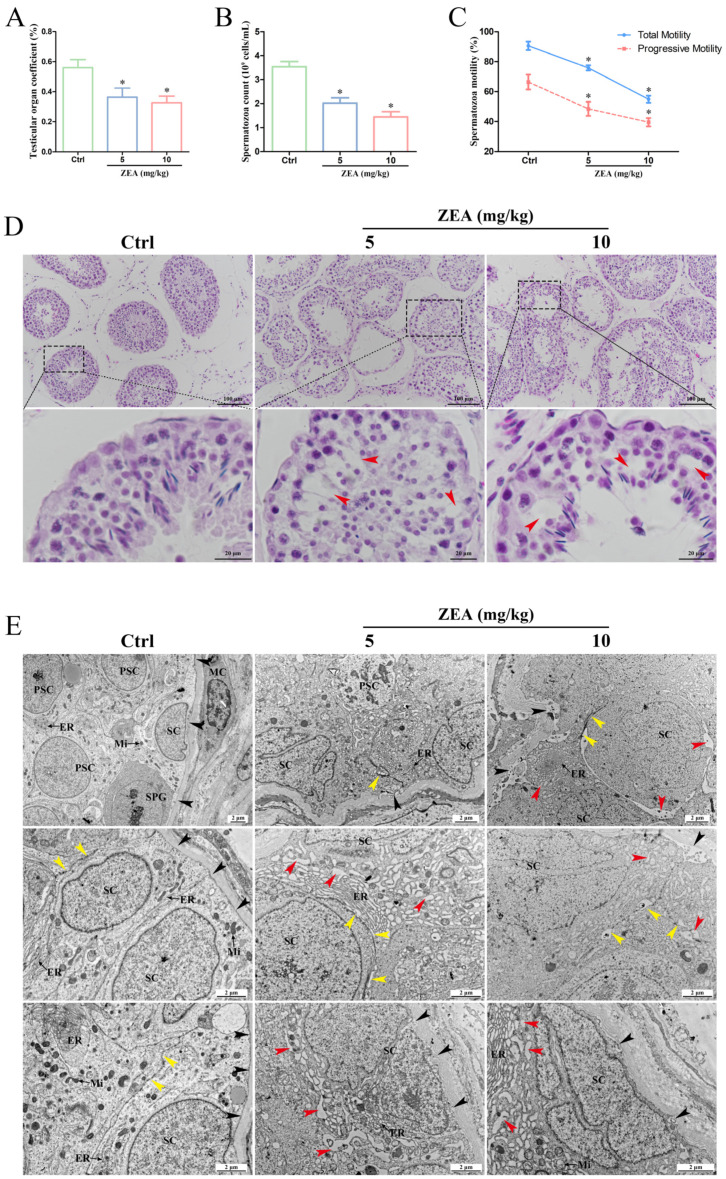
ZEA exposure induces testicular dysfunction in goats. (**A**) Testicular organ coefficient. (**B**) Spermatozoon count. (**C**) The total motility and progressive motility of spermatozoa. (**D**) Histological analysis of testicular tissues stained with H&E. The black dotted boxes are magnified below (scale bars = 20 μm). Red arrowhead: vacuolation in seminiferous tubules. Scale bars = 100 μm. (**E**) Ultrastructure of the testicular germinal epithelium. Black arrowhead: basal lamina of seminiferous tubules. Yellow arrowhead: tight junction. Red arrowhead: intracellular vacuole. MC: myoid cell; SC: Sertoli cell; SPG: spermatogonium; PSC: primary spermatocyte; Mi: mitochondria; ER: endoplasmic reticulum; N: nuclear; Scale bars = 2 μm. * *p* < 0.05 versus the control group (Ctrl).

To investigate the toxic effects of ZEA on the male reproductive function of goats, the relative testis weights and spermatozoon quality were measured after ZEA gavage. The results showed that the testicular organ coefficient was significantly decreased in the 5 mg/kg and 10 mg/kg ZEA groups compared to the control group (*p* < 0.05; Figure 1A). The spermatozoon density was significantly lower in the 5 mg/kg (2.02 × 10^9^ cells/mL) and 10 mg/kg ZEA (1.44 × 10^9^ cells/mL) groups than in the control group (3.54 × 10^9^ cells/mL; *p* < 0.05; Figure 1B). Meanwhile, the total motility and progressive motility of spermatozoa were significantly reduced in the 5 mg/kg and 10 mg/kg ZEA groups (*p* < 0.05; Figure 1C).

The histopathological changes in testes after ZEA treatment were assessed by H&E staining (Figure 1D). In the control group, the seminiferous tubules exhibited a normal structure, and the regular progression of spermatogenesis was observed. Spermatogenic cells and SCs were arranged closely and regularly. After treatment with 5 mg/kg and 10 mg/kg ZEA, atrophy and disorganization of the seminiferous epithelium and abnormal progression of spermatogenesis were observed. In addition, the number of spermatogenic cell layers was decreased, the intercellular space was widened, and the vacuolization (red arrowhead) was evident in most seminiferous tubules.

TEM analysis was performed to further observe the ZEA-induced morphological changes in testes. As shown in Figure 1E, the control group exhibited a normal ultrastructure of seminiferous tubules with regularly arranged spermatogenic cells in various growth cycles and SCs. The convoluted tubules were surrounded by elongated myoid cells and encircled by the regular and continuous basal lamina (black arrowhead). Mitochondria and ER with normal morphology were scattered randomly in the cytoplasm. The TJ (yellow arrowhead) between adjacent SCs was uniform, intact, and arranged linearly. However, in the ZEA treatment groups, the arrangement of spermatogenic epithelial cells was disordered and loose, and vacuoles (red arrowhead) were obviously found. The germinal epithelial cells adjacent to the basal lamina (black arrowhead) were irregular, noncompact, and enfolded, with more vacuoles in junctions. The structure of the BTB was damaged with the local disassembly of TJs, and many vacuoles appeared between adjacent SCs (yellow arrowhead). Remarkably, numerous ER membranes were swollen and dilative, and extensively enlarged and rough ER channels were found within the cytoplasm, which appeared to favor intracellular vacuolar changes (red arrowhead) in SCs.

### 2.2. ZEA Exposure Disrupted BTB Integrity in Goat Testes

**Figure 2:**
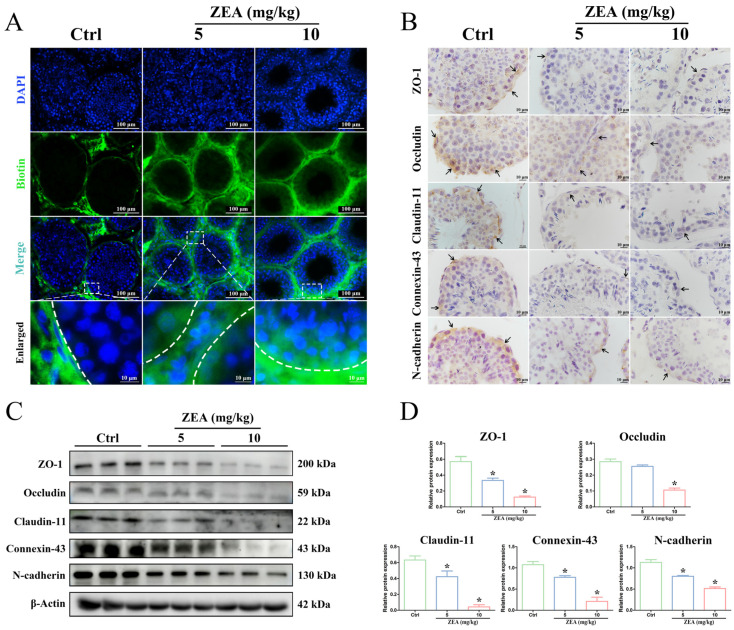
ZEA exposure disrupted BTB integrity in goat testes. (**A**) BTB integrity was determined using a biotin tracer assay. Biotin: green fluorescence. Nuclear (DAPI): blue fluorescence. The basal part of the seminiferous tubules is denoted with a white dotted line. The corresponding scale bars are shown in the bottom right corner. (**B**) The distribution and expression of BTB junctional proteins (ZO-1, Occludin, Claudin-11, Connexin-43, and N-cadherin) were detected by immunohistochemistry. Black arrow: positive staining. Scale bars = 10 μm. (**C**) The expression of BTB junctional proteins (ZO-1, Occludin, Claudin-11, Connexin-43, and N-cadherin) was examined by Western blot. (**D**) Quantitation of the protein expressions of ZO-1, Occludin, Claudin-11, Connexin-43, and N-cadherin. * *p* < 0.05 versus the control group (Ctrl).

To further verify that ZEA exposure disrupted the integrity and function of the BTB, the biotin tracer permeability was assessed in the testes after ZEA gavage (Figure 2A). In the control group, the green fluorescence signal was observed only in the interstitial and basal parts of seminiferous tubules, while the biotin signal across the BTB was not observed in the lumen of the tubules. After treatment with 5 mg/kg and 10 mg/kg ZEA, the green fluorescence signal was found to penetrate the BTB into the lumens of seminiferous tubules, indicating the impairment of BTB integrity.

The localization and expression of junctional proteins are usually used to evaluate the integrity of the BTB structure [16]. In this study, IHC analysis was performed to detect changes in the distribution and expression of critical proteins (Figure 2B), including TJ proteins (ZO-1, Occludin, and Claudin-11), GJ protein (Connexin-43), and ES protein (N-cadherin). In the control group, the positive immunoreactions of ZO-1, Occludin, Claudin-11, Connexin-43, and N-cadherin proteins were predominantly detected along the circumference near the basal region of the seminiferous tubules between SCs and spermatogenic cells (black arrow). After ZEA exposure, the positive staining of these proteins decreased dramatically, although the protein expression localization was identical to that in the control group. Furthermore, the expression levels of these BTB junctional proteins were verified by Western blot analysis (Figure 2C,D). Consistent with the IHC results, the protein levels of ZO-1, Claudin-11, Connexin-43, and N-cadherin were significantly decreased after ZEA exposure at 5 mg/kg and 10 mg/kg compared to the control group (*p* < 0.05). In addition, a significantly reduced expression of Occludin protein was detected in the 10 mg/kg ZEA group compared to the control group (*p* < 0.05). These results revealed that ZEA exposure evidently disrupted the integrity of the BTB in goat testes.

### 2.3. ZEA Decreased SC Viability and Induced BTB Damage In Vitro

**Figure 3:**
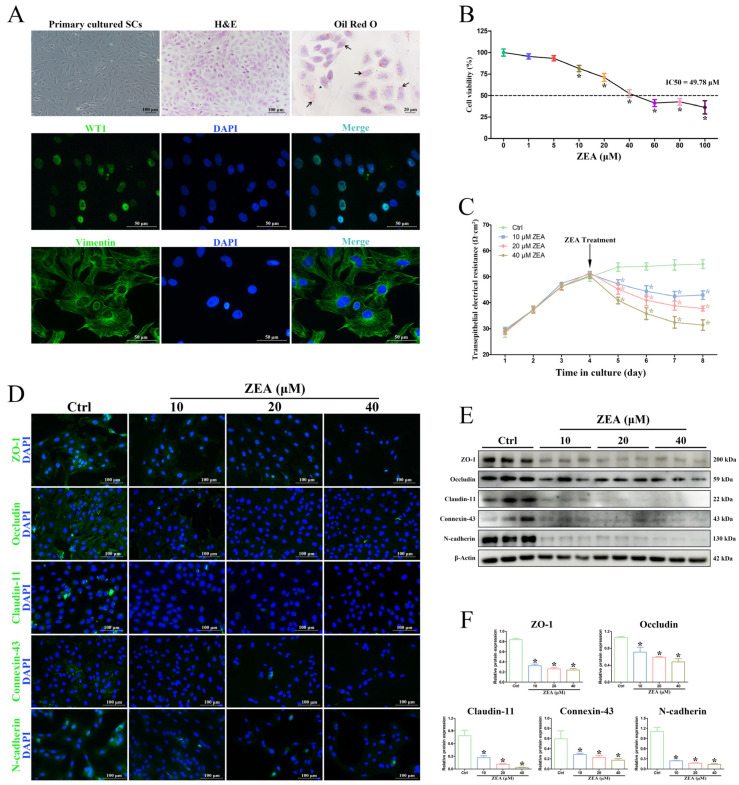
ZEA decreased the viability of SCs and induced BTB damage in vitro. (**A**) Identification of isolated primary SCs on Day 4 by morphology observation, H&E staining, Oil Red O staining, and immunofluorescence staining for the SC markers WT1 and Vimentin. Black arrow: red lipid droplets. WT1 and Vimentin: green fluorescence. Nuclear (DAPI): blue fluorescence. The corresponding scale bars are shown in the bottom right corner. (**B**) Cell viability was assayed using CCK-8. SCs were exposed to different concentrations of ZEA for 24 h. (**C**) The transepithelial electrical resistance of SCs in the control and ZEA groups. The arrow marks the time of ZEA treatment. (**D**) The distribution and expression of BTB junctional proteins (ZO-1, Occludin, Claudin-11, Connexin-43, and N-cadherin) in SCs were detected by immunofluorescence. ZO-1, Occludin, Claudin-11, Connexin-43, and N-cadherin: green fluorescence. Nuclear (DAPI): blue fluorescence. Scale bars = 100 μm. (**E**) The expression of BTB junctional proteins (ZO-1, Occludin, Claudin-11, Connexin-43, and N-cadherin) in SCs was examined by Western blot. (**F**) Quantitation of the protein expressions of ZO-1, Occludin, Claudin-11, Connexin-43, and N-cadherin. * *p* < 0.05 versus the control group (Ctrl).

Primary SCs were isolated from goat testes and used for the in vitro ZEA treatment to validate the adverse impact of ZEA on BTB integrity. The morphology and purity of SCs were identified by H&E staining, Oil red O staining, and IF staining (Figure 3A). The staining results of the specific SC markers WT1 and Vimentin confirmed that the successfully isolated SCs had high purity. The obtained goat SCs were exposed to different concentrations of ZEA for 24 h, and then the cell viability was determined by CCK-8 (Figure 3B). SC viability showed a dose-dependent decrease, and cell viability was significantly reduced when the concentration of ZEA was 10 μM or higher (*p* < 0.05). The IC50 of SCs exposed to ZEA was under the concentration of 49.78 μM, and thus, exposure concentrations of ZEA of 10, 20, and 40 μM were selected for subsequent experiments.

The damage to BTB permeability after ZEA treatment was analyzed by measuring the TER daily. The results showed that treatment with 10, 20, and 40 μM ZEA induced a significant decrease in the level of TER compared to the control group (*p* < 0.05) in a dose-dependent manner (Figure 3C). Furthermore, the localization and expression analyses of BTB functional proteins in SCs showed that the fluorescence intensities of the ZO-1, Occludin, Claudin-11, Connexin-43, and N-cadherin protein expressions were significantly decreased after ZEA treatment, while the expression localization of these proteins was not different from that of the control group (Figure 3D), which was in agreement with the results after the in vivo ZEA gavage. Western blot analyses also revealed a significant decrease in the levels of ZO-1, Occludin, Claudin-11, Connexin-43, and N-cadherin proteins after ZEA treatment (*p* < 0.05; Figure 3E,F). These results implied that ZEA treatment could lead to the junctional function impairment of goat SCs in vitro.

### 2.4. ZEA Treatment Induced Cytoplasmic Vacuolation in SCs

**Figure 4:**
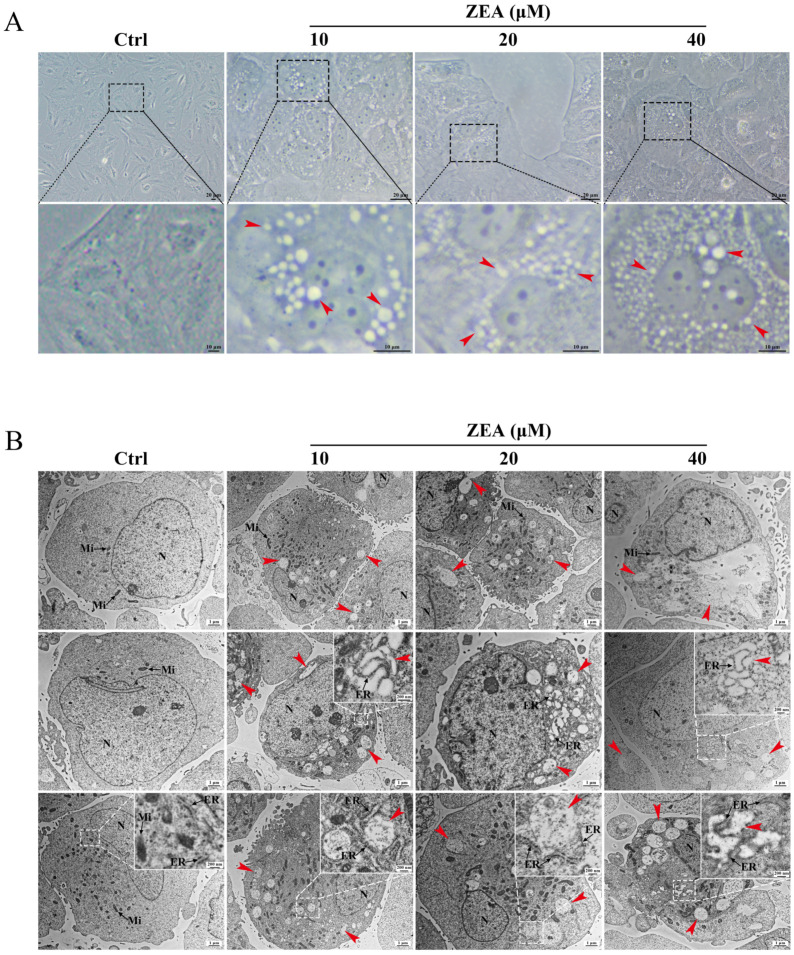
ZEA treatment induced cytoplasmic vacuolation in SCs. (**A**) ZEA-treated SCs were observed by light microscopy. The black dotted boxes are magnified below (scale bars = 10 μm). Red arrowhead: cytoplasmic vacuoles. Scale bars = 20 μm. (**B**) ZEA-treated SCs were observed by transmission electron microscopy. The white dotted boxes are magnified in the upper right inset (scale bars = 200 nm). Red arrowhead: intracellular vacuoles. Mi: mitochondria; ER: endoplasmic reticulum; N: nuclear; Scale bars = 1 μm.

The morphological changes in SCs after ZEA treatment were investigated by light microscopy. The results showed that ZEA exposure caused the formation and accumulation of massive cytoplasmic vacuoles (red arrowhead) in the vicinity of the SC nucleus (Figure 4A). Vacuoles were obviously observed in the 10 μM and 20 μM ZEA-treated groups and were even more conspicuous and widely distributed in the SCs after the 40 μM ZEA treatment.

Furthermore, the ZEA-induced cytoplasmic vacuolization was further examined by TEM (Figure 4B). In the control group, no obvious vacuole formation was observed in the cytoplasm, and the morphology of SCs was normal: the mitochondria displayed double membranes, mitochondrial cristae were dense and visible, and the dense cytoplasm contained abundant and intact ER. After ZEA treatment, a large number of single-membrane cytoplasmic vacuoles (red arrowhead) were clearly observed, and no cytoplasmic material was found within these vacuoles. The structure of mitochondria appeared almost normal, and the vacuoles appeared to not colocalize with mitochondria. Remarkably, part of the ER structure exhibited the disorganization, expansion, and loss of ribosomes and was contiguous with the detected vacuoles in the cytoplasm. The ultrastructural changes in the ER of SCs were consistent with the in vivo results in Figure 1E, indicating that the cytoplasmic vacuoles induced by ZEA may originate from ER membranes. Interestingly, these morphological changes in SCs matched well with the features of paraptosis, in which paraptotic cell death is mainly characterized by cytoplasmic vacuolation arising from ER dilation [25,27].

### 2.5. ZEA Exposure Triggered SC Paraptosis

**Figure 5:**
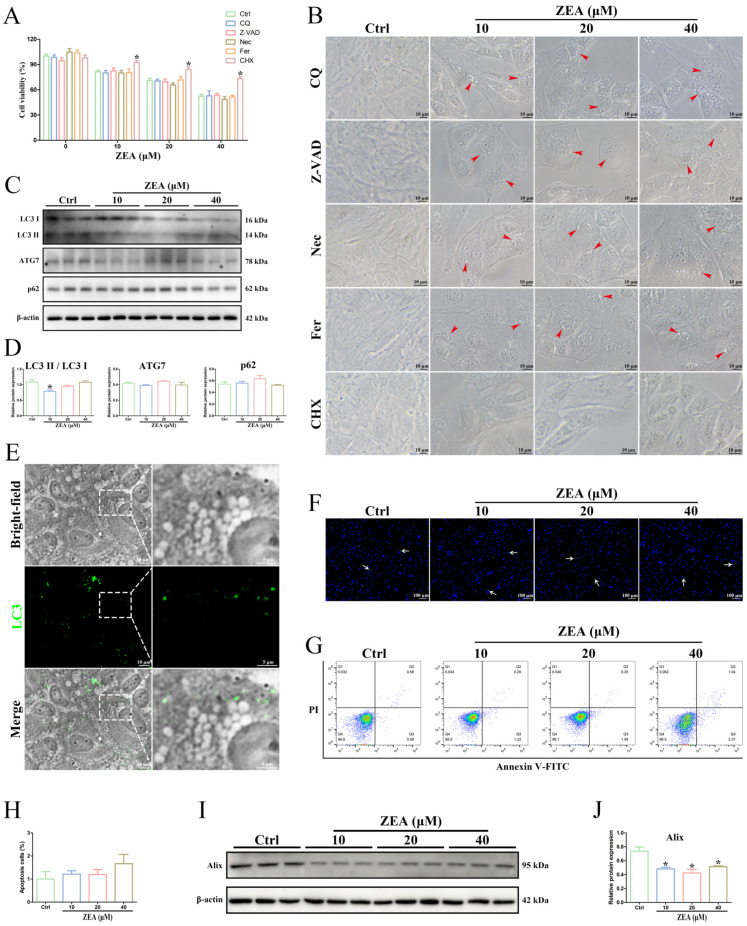
ZEA exposure triggered SC paraptosis. (**A**) Cellular viability was assessed using a CCK-8 assay. (**B**) Cellular morphologies were observed by light microscopy. Red arrowhead: cytoplasmic vacuoles. SCs were pretreated with 25 μM Chloroquine, 25 μM Z-VAD-FMK, 20 μM Necrostatin-1, 10 μM Ferrostatin-1, and 5 μM Cycloheximide, respectively, followed by ZEA treatment. CQ: Chloroquine; Z-VAD: Z-VAD-FMK; Nec: Necrostatin-1; Fer: Ferrostatin-1; and CHX: Cycloheximide. (**C**) The expression of autophagic markers (LC3, ATG7, and p62) in SCs was examined by Western blot. (**D**) Quantitation of the protein expressions of LC3II/LC3I, ATG7, and p62. (**E**) The distribution of LC3 in SCs was detected by immunofluorescence. SCs were exposed to 40 μM ZEA for 24 h. The white dotted boxes are magnified on the right (scale bars = 5 μm). LC3: green fluorescence. Nuclear (DAPI): blue fluorescence. Scale bars = 10 μm. (**F**) The nuclear morphology of SCs was measured by Hoechst 33342 staining. White arrow: apoptotic cells (nuclei with high intense blue fluorescence). Scale bars = 100 μm. (**G**,**H**) Apoptosis was measured by Annexin V-FITC/PI flow cytometry analysis. (**I**) The expression of the BTB functional protein Alix in SCs was examined by Western blot. (**J**) Quantitation of Alix protein expression. * *p* < 0.05 versus the control group (Ctrl).

To examine whether the vacuoles that occurred after ZEA treatment were related to the autophagic process, the autophagy inhibitor Chloroquine was used to interfere with autophagy in SCs. Pretreatment with Chloroquine failed to inhibit ZEA-induced cell death and vacuolation in SCs (Figure 5A,B). Moreover, the Western blot analysis showed that the protein levels of the autophagic markers ATG7 and p62 after ZEA treatment were not significantly different from those in the control group (*p* > 0.05; Figure 5C,D). The ratio of LC3II/LC3I was reduced only in the 10 μM ZEA groups (*p* < 0.05; Figure 5C,D), suggesting a slightly disturbed autophagy in ZEA-treated SCs. However, the immunodetection of LC3 was not colocalized with the cytoplasmic vacuoles in SCs after the 40 μM ZEA treatment (Figure 5E). Remarkably, ZEA-induced cytoplasmic vacuoles in SCs with single-membrane structures did not exhibit the typical morphological features of autophagosomes (Figure 4B), which contained fragments of cellular organelles and had double-membrane structures [39]. These findings indicated that autophagy was not a potential reason for ZEA-induced cellular vacuolation in SCs.

To further investigate the possible involvement of apoptosis in ZEA-induced cell death, SCs were pretreated with the apoptotic inhibitor Z-VAD-FMK. The addition of Z-VAD-FMK did not significantly alleviate ZEA-induced cell death and did not block the formation of cytoplasmic vacuoles in SCs (Figure 5A,B). Furthermore, the results of Hoechst 33342 staining and Annexin V-FITC/PI double-staining showed that only a very small proportion of apoptotic cells was detected in the ZEA-treated groups (Figure 5F–H), while there was no significant difference from the control group (*p* > 0.05). In addition, ultrastructural characteristics of apoptosis, such as cellular shrinkage, plasma membrane blebbing, nuclear fragmentation, chromatin condensation, and apoptotic body formation [40], were not observed in ZEA-treated SCs (Figure 4B). These results suggested that ZEA-induced SC death did not correspond to the conventional apoptotic process.

Meanwhile, the impacts of the necroptosis inhibitor Necrostatin-1 and ferroptosis inhibitor Ferrostatin-1 on cell viability and vacuole formation were investigated in ZEA-treated SCs. As expected, treatment with neither Necrostatin-1 nor Ferrostatin-1 significantly decreased ZEA-induced SC death or the number of vacuoles (*p* > 0.05; Figure 5A,B).

Furthermore, treatment with the protein synthesis inhibitor CHX, which specifically inhibited paraptosis, was performed to detect the effects on ZEA-induced SC death. The results showed that SC death and the number of intracellular vacuoles were significantly attenuated by the CHX treatment (*p* < 0.05; Figure 5A,B), indicating that the inhibition of paraptosis could mitigate ZEA-induced damage to SCs. Subsequently, to confirm the ZEA-induced paraptotic cell death in SCs, the expression level of Alix protein, which is a known endogenous inhibitor of paraptosis [26,41], was detected by Western blot. As shown in Figure 5I,J, the ZEA treatment significantly decreased the protein expression of Alix (*p* < 0.05). These findings confirmed that the structural and functional destruction of SCs induced by ZEA was closely associated with paraptotic cell death.

### 2.6. ZEA-Induced Paraptosis Was Accompanied by ER Stress in SCs

**Figure 6:**
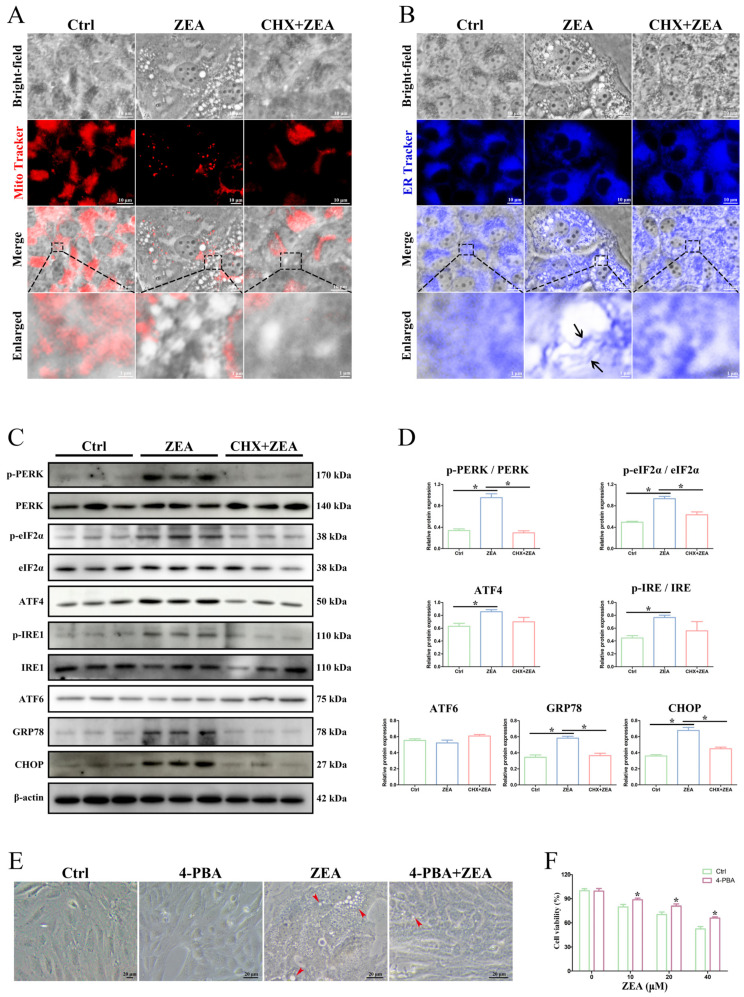
ZEA-induced paraptosis was accompanied by ER stress in SCs. SCs were stained with MitoTracker Red (**A**) and ER-Tracker Blue (**B**) and observed under a fluorescence microscope. SCs were pretreated with 5 μM CHX, followed by 40 μM ZEA treatment. MitoTracker Red: red fluorescence. ER-Tracker Blue: blue fluorescence. Black arrow: ER dilation. CHX: Cycloheximide. The corresponding scale bars are shown in the bottom right corner. (**C**) The expressions of ER stress marker proteins (p-PERK, PERK, p-eIF2α, eIF2α, ATF4, p-IRE1, IRE1, ATF6, GRP78, and CHOP) in SCs were examined by Western blot. SCs were pretreated with 5 μM CHX, followed by 40 μM ZEA treatment. (**D**) Quantitation of the protein expressions of p-PERK/PERK, p-eIF2α/eIF2α, ATF4, p-IRE1/IRE1, ATF6, GRP78, and CHOP. p-PERK: phosphorylated PERK; p-eIF2α: phosphorylated eIF2α; and p-IRE1: phosphorylated IRE1. (**E**) Cellular morphologies were observed by light microscopy. Red arrowhead: cytoplasmic vacuoles. SCs were pretreated with 1 mM 4-phenylbutyric acid, followed by 40 μM ZEA treatment. Scale bars = 20 μm. (**F**) Cellular viability was assessed using CCK-8 assay. SCs were pretreated with 1 mM 4-phenylbutyric acid, followed by ZEA treatment. 4-PBA: 4-phenylbutyric acid. * *p* < 0.05.

To detect whether ZEA-induced paraptosis-related cytoplasmic vacuoles arose from mitochondria or ER, the mitochondria fluorescent probe (MitoTracker Red) and ER fluorescent probe (ER-Tracker Blue) were used to stain the ZEA-treated SCs (Figure 6A,B). The results showed that the membrane of vacuoles induced by ZEA was colocalized with the fluorescence of ER-Tracker Blue (Figure 6B), while it was not stained by MitoTracker Red (Figure 6A). In addition, the CHX pretreatment significantly mitigated ZEA-induced paraptosis-related ER dilation (Figure 6B). These findings further indicated that the ZEA-induced vacuoles were derived from the dilated ER.

Expression analysis of the ER stress marker proteins, including pancreatic ER kinase (PKR)-like ER kinase (PERK), translation initiation factor 2α (eIF2α), activating transcription factor 4 (ATF4), inositol-requiring enzyme-1 (IRE1), activating transcription factor 6 (ATF6), glucose-regulating protein 78 (GRP78), and C/EBP homologous protein (CHOP), was performed to validate the ER stress that mediated paraptosis under ZEA exposure (Figure 6C,D). ZEA treatment caused the significant upregulation of phosphorylated PERK, phosphorylated eIF2α, ATF4, phosphorylated IRE1, GRP78, and CHOP along with the occurrence of paraptosis in SCs (*p* < 0.05), while the expression of ATF6 had no significant change (*p* > 0.05). In addition, CHX pretreatment in ZEA-exposed SCs significantly decreased the protein levels of phosphorylated PERK, phosphorylated eIF2α, GRP78, and CHOP (*p* < 0.05), indicating that ZEA-induced ER stress was alleviated by the inhibition of paraptosis. Moreover, ZEA-induced cytoplasmic vacuolation and SC death were remarkably alleviated by treatment with ER stress inhibitor 4-phenylbutyric acid (Figure 6E,F). In general, ZEA-induced paraptosis is accompanied by ER stress in SCs.

## 3. Discussion

ZEA is one of the most prevalent mycotoxins and is present in a wide range of animal feeds [3,4,5]. The estrogen-like activity of ZEA confers its high male reproductive toxicity, leading to spermatogenesis dysfunction and male sterility [4,17]. The pathogenesis of ZEA in the male reproductive system is primarily focused on its adverse impacts on germ cells and testicular structure [7,17]. Studies have found that 20 µg/kg ZEA exposure disrupts the spermatogenesis process and causes a decrease in the concentration, motility, and viability of mouse spermatozoa [42]. A similar report by Gao et al. [43] also suggested that a significant decrease in spermatozoon concentration and motility was detected in 20 µg/kg ZEA-treated mice, as well as a diminished testis index and abnormal spermatozoon structure. In this study, ZEA-induced reproductive damage in male goats was validated by the in vivo ZEA gavage. The testicular organ coefficient, spermatozoon concentration, and the total motility and progressive motility of spermatozoa were significantly reduced after ZEA treatment, implying impaired spermatogenesis in goats. Further histopathological and ultrastructural observation found that ZEA exposure induced damage to the seminiferous tubules, disruption of the BTB structure, the occurrence of vacuolations, and swollen and dilative ER in SCs. These expected morphological changes in the testes are fundamental to intensively exploring the potential toxicity mechanism of ZEA-induced reproductive disorders in male goats.

The BTB is a special barrier structure for the male reproductive system and poses a tight obstacle to preventing the invasion of harmful substances and building a condign biochemical and immunological microenvironment [15,16]. The integrity of the BTB is responsible for ensuring normal testicular function and spermatozoon development and is considered to be an important pathway in preventing reproductive toxicity [15,44]. Many reproductive toxicants mainly target the BTB structure by downregulating the expression of junction proteins, such as TJ proteins, GJ proteins, and ES proteins [17,18,22]. Moreover, the destruction of male reproductive function induced by ZEA has been extensively investigated and is mostly attributed to the disruption of the BTB integrity [17,24]. Previous studies have shown that intraperitoneal injection of ZEA significantly decreased the quality of spermatozoa in Kunming mice and the protein expression of N-cadherin, Vimentin, and Claudin-11, which are related to the BTB [23]. Another study by She et al. [22] also found that ZEA exposure destroyed the BTB structure and decreased the expression of junctional proteins, resulting in male reproductive injury in mice. As expected, our study found impaired BTB integrity by ZEA treatment by using a biotin tracer to assess the BTB permeability in goat testes. Moreover, both IHC and Western blot analyses showed a decreased expression of BTB junction proteins in ZEA-treated testicular tissue, which was consistent with previous reports in mice [22,23]. In addition, considerable studies have also indicated that ZEA treatment, when in vitro, can hinder the survival of SCs and destroy the structure of the BTB through different signaling pathways to cause testicular toxicity [13,24,45,46]. In mammalian testes, SCs are the major somatic cells of the seminiferous tubules and play crucial roles in spermatogenesis and the regulation of testicular function [47,48]. The most prominent contribution of SCs in testicular tissue is to establish the BTB [15,16]. In the current study, primary SCs isolated from goats were used to further validate the toxic effects of ZEA on the structure and integrity of the BTB. We found that ZEA exposure induced dose-dependent decreases in cell viability and TER in SCs. A significant decrease in the expression of junction proteins, including ZO-1, Occludin, Claudin-11, Connexin-43, and N-cadherin, was also detected in ZEA-treated goat SCs and was consistent with the results in ZEA-treated testes in vivo, indicating that ZEA seriously disrupted BTB permeability in goats. Remarkably, further morphological analysis revealed that ZEA treatment caused the formation of massive vacuoles in the cytoplasm of SCs. More importantly, these vacuoles appeared to be contiguous with swollen and dilative ER membranes. These findings supported the important hypothesis that the occurrence of vacuolations arising from ER dilation may be in accord with the typic features of paraptosis, which provided valuable references for understanding the underlying mechanism of ZEA-induced BTB damage in goats.

It is widely recognized that ZEA-induced damage to the reproductive system is inevitably involved in different types of cell death [4]. Apoptosis and autophagy are two classical pathways of PCD, based on their specific morphological criteria and have been well described to contribute to ZEA-induced reproductive toxicity [13,22,37]. Previous studies have shown that ZEA-induced cytotoxic injury in piglet SCs was coincident with cell apoptosis [49], and ZEA exposure-induced male reproductive toxicity in dairy goats was related to oxidative stress and autophagy in SCs [13]. However, in this study, ZEA-induced SC damage was not eliminated by the inhibition of both apoptosis and autophagy. The typical morphological features of apoptosis, such as nuclear fragmentation, chromatin condensation, and apoptotic body formation, were not observed in ZEA-treated SCs. Only a very small proportion of apoptotic cells were detected by Hoechst staining and Annexin V-FITC/PI double-staining after ZEA treatment, but there was no significant difference from the control group. Furthermore, the double-membrane autophagosomes wrapping the undegraded substances that indicated autophagy were also not found in ZEA-treated SCs. Although a slight decrease in the ratio of LC3II/LC3I was detected after ZEA treatment, the immunodetection of LC3 was not colocalized with the cytoplasmic vacuoles. In addition, treatment with the necroptosis inhibitor Necrostatin-1 and ferroptosis inhibitor Ferrostatin-1 did not affect ZEA-induced cell death or the number of vacuoles. Interestingly, our results revealed different morphological and ultrastructural alterations to apoptosis and autophagy in ZEA-treated SCs: the presence of single-membrane cytoplasmic vacuoles with highly swollen ER. These findings further implied that ZEA-induced cytoplasmic vacuolation in goat SCs was attributed to the other types of cell death.

Paraptosis is a recently defined caspase-independent process of PCD that differs from classical apoptosis by lacking the morphological hallmarks of apoptosis [27,41]. The main feature of paraptosis is the occurrence of extensive cytoplasmic vacuolization along with the swelling of ER and/or mitochondria [25,27]. The process of paraptosis requires new protein synthesis and is specifically inhibited by the protein synthesis inhibitor CHX [25,27]. The addition of CHX has been reported to reverse paraptosis triggered by different inducers [35,50,51]. Moreover, the molecular marker Alix protein, as a negative regulator of paraptosis, is usually used to assess paraptosis cell death [26,41]. In the present study, an ultrastructural observation revealed the possible implication of paraptosis in ZEA-induced vacuole generation and damaged ER membranes. Furthermore, the formation of cytoplasmic vacuoles induced by ZEA was found to be inhibited by CHX treatment, and the expression level of Alix protein was significantly reduced after ZEA treatment. The above results indicate that paraptosis favors ZEA-induced SC dysfunction in goats and may be accompanied by ER stress. However, the regulatory mechanism of paraptosis triggered by ZEA is still largely unclear.

It is well known that ER stress is generally closely associated with the occurrence of paraptosis [35,52]. Our results showed that mitochondria in SCs exhibited normal morphology after ZEA treatment, and the vacuoles induced by ZEA could not be marked by a mitochondrial fluorescent probe, which suggested that cytoplasmic vacuolation may be unrelated to mitochondria. Most notably, the ZEA-induced vacuoles were marked by a specific ER fluorescent probe and colocalized well with the dilative ER, which confirmed that the cytoplasmic vacuoles arose from ER swelling. Further pretreatment with CHX significantly inhibited the ZEA-induced vacuolar expansion of the ER, and pretreatment with the ER stress inhibitor 4-phenylbutyric acid significantly decreased ZEA-induced vacuoles and SC death, indicating the direct relevance of ER stress to paraptosis. The ER mainly functions in protein synthesis, which is necessary for paraptosis induction [27,28]. The accumulation of unfolded and misfolded proteins in the ER lumen can cause ER stress and trigger the unfolded protein response (UPR), which is considered to be a protective mechanism to maintain cellular homeostasis [29,53]. The UPR involves the activation of signaling pathways, including PERK, IRE1, and ATF6 [54]. The eIF2α phosphorylation is induced by autophosphorylated PERK during ER stress, which then triggers the expression of ATF4 [55,56]. In addition, GRP78 (protein-folding chaperone protein) and CHOP, as ER stress sensor molecules, exhibit upregulated expression during ER stress [57,58]. The current study indicated that the expression of critical ER stress marker proteins (phosphorylated PERK, phosphorylated eIF2α, ATF4, phosphorylated IRE1, GRP78, and CHOP) was significantly increased after ZEA treatment, indicating the ZEA-induced activation of ER stress. Moreover, the levels of phosphorylated PERK and phosphorylated eIF2α, which are involved in the activation of the PERK signaling pathway, were significantly decreased under CHX-mediated paraptosis inhibition. Evidence has highlighted that PERK signaling pathway-mediated ER stress can regulate paraptosis, which participates in the process of cell death, such as in melanoma and glioblastoma cells [31,59]. In light of the above considerations, the ER stress-related PERK signaling pathway may play a vital role in ZEA-induced paraptosis in goat SCs.

## 4. Materials and Methods

### 4.1. Animals and Experimental Design

All procedures involving animals were approved by the Institutional Animal Care and Use Committee of Northwest A&F University (Approval code: DY2023033), Shaanxi, China. Fifteen healthy male Guanzhong goats (*Capra hircus*; approximately 1–2 years of age) weighing 30–40 kg in body weight were used in this study. All goats were fed and managed continually according to the management standards of goats. After the adaptation period of two weeks, the goats were randomly divided into three groups: the control and two ZEA-treatment groups (n = 5 per group). The ZEA-treatment groups were administered ZEA (5 mg/kg/day or 10 mg/kg/day) dissolved in corn oil as the vehicle by gavage for 21 d. The control group was administered only the vehicle. The ZEA was purchased from Sigma-Aldrich (Cat# Z2125, St. Louis, MO, USA). Doses and administration schedules for ZEA were taken based on the preliminary results from our laboratory. Forty-eight hours after the last gavage, semen samples were collected from each goat using an artificial vagina. Subsequently, all animals were euthanized with sodium pentobarbital (50 mg/kg), and the testes were immediately removed. The left testes of each goat were employed for hematoxylin–eosin (H&E) staining, ultrastructural observation, BTB integrity detection, and immunohistochemistry (IHC) analysis. The right testes were stored in liquid nitrogen for Western blot analysis.

### 4.2. Evaluation of Testicular Organ Coefficients

All goats were weighed before anesthesia, and bilateral testicles were immediately removed and weighed after anesthesia. The testicular organ coefficient was calculated as the formula: Bilateral testicular weight/body weight × 100%.

### 4.3. Spermatozoon Quality Analysis

The spermatozoon number and motility analysis were evaluated using the computer-assisted sperm analysis (CASA) system (Hamilton Thorne Research, Beverly, CA, USA) according to a reported procedure [60]. All the semen samples were observed under a phase-contrast microscope (Nikon, Tokyo, Japan).

### 4.4. Hematoxylin–Eosin (H&E) Staining

The testes and SCs were fixed in 4% paraformaldehyde for 48 h, followed by dehydration and paraffin embedding. The embedded tissues were sectioned to 5 μm thickness, deparaffinized and rehydrated, and then stained with H&E for light microscopy observation (Nikon, Tokyo, Japan).

### 4.5. Transmission Electron Microscopy (TEM) Analysis

The testes and SCs were fixed in 2.5% glutaraldehyde and post-fixed in 1% osmic acid. After being dehydrated and embedded in epoxy resin, ultrathin sections were prepared and stained with uranyl acetate and citrate. The sections were examined with a Tecnai G2 Spirit Bio-Twin transmission electron microscope (FEI Company, Hillsboro, OR, USA).

### 4.6. BTB Integrity Assay

The integrity of the BTB was assessed by using a biotin tracer, as described previously [61]. Briefly, 100 μL of EZ-Link^TM^ Sulfo-NHS-LC-Biotin (10 mg/mL in PBS containing 1 mM CaCl_2_; Cat# 21335, Thermo Fisher Scientific, Waltham, MA, USA) was injected into the testis interstitium. After 30 min, the testes were cut into 10 μm cryosections and fixed in 4% paraformaldehyde for 15 min. The sections were blocked with 5% fetal bovine serum for 1 h and then incubated with FITC-labeled streptavidin (Cat# S3762, Sigma-Aldrich, St. Louis, MO, USA) for 30 min at room temperature. Finally, the sections were added to the mounting medium with DAPI (Cat# ab104139, Abcam, Cambridge, MA, USA) and visualized by fluorescence microscopy (Olympus BX53, Tokyo, Japan).

### 4.7. Immunohistochemistry (IHC) Staining

The IHC experiment was performed as described previously [62]. Briefly, testes sections (6 μm) were dewaxed with xylene and gradient ethanol consecutively, followed by antigen retrieval. The sections were incubated with 0.5% bovine serum albumin for 1 h and then incubated overnight with the following antibodies: anti-ZO-1 (1:100, Cat# ab276131, Abcam, Cambridge, MA, USA), anti-Occludin (1:200, Cat# ab216327, Abcam, Cambridge, MA, USA), anti-Claudin-11 (1:100, Cat# 36-4500, Thermo Fisher Scientific, Waltham, MA, USA), anti-Connexin-43 (1:500, Cat# ab217676, Abcam, Cambridge, MA, USA), and anti-N-cadherin (1:500, Cat# ab207608, Abcam, Cambridge, MA, USA). The sections were incubated for 1 h with HRP-labeled secondary antibody (1:2000, Cat# ab205722, Abcam, Cambridge, MA, USA), followed by DAB staining (Cat# ab64264, Abcam, Cambridge, MA, USA) and observation under a microscope (Nikon, Tokyo, Japan).

### 4.8. Western Blot Analysis

RIPA lysis buffer (Cat# R0010, Solarbio, Beijing, China) containing phenylmethylsulfonyl fluoride (PMSF), phosphatase inhibitors, and protease inhibitors was used for protein extraction. The protocol of Western blot was performed according to a reported method [63]. Primary antibody: anti-ZO-1 (1:1000, Cat# ab276131, Abcam, Cambridge, MA, USA), anti-Occludin (1:1000, Cat# ab216327, Abcam, Cambridge, MA, USA), anti-Claudin-11 (1:300, Cat# 36-4500, Thermo Fisher Scientific, Waltham, MA, USA), anti-Connexin-43 (1:1000, Cat# ab217676, Abcam, Cambridge, MA, USA), anti-N-cadherin (1:1000, Cat# ab207608, Abcam, Cambridge, MA, USA), anti-LC3B (1:1000, Cat# ab192890, Abcam, Cambridge, MA, USA), anti-ATG7 (1:5000, Cat# ab133528, Abcam, Cambridge, MA, USA), anti-p62 (1:5000, Cat# ab109012, Abcam, Cambridge, MA, USA), anti-Alix (1:1000, Cat# 92880, Cell Signaling Technology, Boston, MA, USA), anti-p-PERK (1:1000, Cat# 3179, Cell Signaling Technology, Boston, MA, USA), anti-PERK (1:1000, Cat# 3192, Cell Signaling Technology, Boston, MA, USA), anti-p-eIF2α (1:1000, Cat# 3398, Cell Signaling Technology, Boston, MA, USA), anti-eIF2α (1:1000, Cat# 5324, Cell Signaling Technology, Boston, MA, USA), anti-ATF4 (1:1000, Cat# ab270980, Abcam, Cambridge, MA, USA), anti-p-IRE1 (1:2000, Cat# ab124945, Abcam, Cambridge, MA, USA), anti-IRE1 (1:1000, Cat# ab37073, Abcam, Cambridge, MA, USA), anti-ATF6 (1:1000, Cat# ab37149, Abcam, Cambridge, MA, USA), anti-GRP78 (1:1000, Cat# ab21685, Abcam, Cambridge, MA, USA), anti-CHOP (1:1000, Cat# 5554, Cell Signaling Technology, Boston, MA, USA), and anti-β-actin (1:1000, Cat# ab8227, Abcam, Cambridge, MA, USA). The density of the protein bands was analyzed using Quantity One software (Bio-Rad, Richmond, VA, USA).

### 4.9. Isolation, Culture, and Identification of Goat SCs

Primary goat SCs were isolated from 3-month-old goats, according to a previous study [64]. In brief, the testes were minced into small pieces of 1 mm^3^ and digested for 30 min in PBS containing 1 mg/mL collagenase IV (Cat# C4-BIOC, Sigma-Aldrich, St. Louis, MO, USA) in a 37 °C water bath. The supernatant was discarded, and 0.25% trypsin (Cat# T1300, Solarbio, Beijing, China) was added to cover the tissue and digested for 15 min in a 37 °C water bath. After the digestion was stopped, the mixture was passed through a 200 μm stainless mesh and resuspended in DMEM/F-12 (Cat# 11320033, Gibco, Grand Island, NE, USA) containing 10% fetal bovine serum (Cat# 16140071, Gibco, Grand Island, NE, USA) and antibiotics (50 IU/mL penicillin, 50 mg/mL streptomycin). The SC density was adjusted and grown at 35 °C in 5% CO_2_. During the culture, the samples were washed with fresh culture medium to remove the remaining germ cells. The obtained SCs were identified by morphology observation, H&E staining, Oil Red O staining, and immunofluorescence (IF) staining for the cell markers WT1 and Vimentin.

### 4.10. Cell Treatment

ZEA, Chloroquine (Cat# C6628, Sigma-Aldrich, St. Louis, MO, USA), Z-VAD-FMK (Cat# V116, Sigma-Aldrich, St. Louis, MO, USA), Necrostatin-1 (Cat# N9037, Sigma-Aldrich, St. Louis, MO, USA), Ferrostatin-1 (Cat# SML0583, Sigma-Aldrich, St. Louis, MO, USA), CHX (Cat# 239763-M, Sigma-Aldrich, St. Louis, MO, USA), and 4-phenylbutyric acid (Cat# P21005, Sigma-Aldrich, St. Louis, MO, USA) were dissolved in DMSO to obtain a stock solution, and diluted in the cell culture medium to produce working concentrations. To evaluate the cytotoxicity of ZEA, SCs were exposed to different concentrations of ZEA (0, 1, 5, 10, 20, 40, 60, 80, and 100 μM) on the fourth day for 24 h. For the intervention experiments, SCs were pretreated with 25 μM Chloroquine, 25 μM Z-VAD-FMK, 20 μM Necrostatin-1, 10 μM Ferrostatin-1, 5 μM CHX, and 1 mM 4-phenylbutyric acid for 4 h, respectively, and then were treated with ZEA (10, 20, 40 μM) for 24 h.

### 4.11. Cell Viability Assay

Cell viability was assessed using a Cell Counting Kit-8 (CCK-8) (Cat# CA1210, Solarbio, Beijing, China). In brief, SCs were plated at a density of 1 × 10^4^ cells per well in 96-well plates. After the indicated treatment, the 10% CCK-8 solution (diluted with DMEM/F-12 medium) was added to each well and incubated at 37 °C for 2 h. The absorbance was measured at 450 nm by using a microplate reader (Bio-Rad 680, Richmond, VA, USA).

### 4.12. Transepithelial Electrical Resistance (TER) Measurement

The permeability of the BTB in vitro was detected by TER, as described previously [65]. Briefly, SCs (1 × 10^6^ cells/cm^2^) were seeded in a transwell chamber (Cat# PTHT24H48, Millipore, Bedford, TX, USA) and cultured to allow BTB assembly. The experiment lasted 8 d, and the medium was renewed daily. In the whole process, the TER in each unit was recorded daily according to the Millicell ERS system (Millipore, Bedford, TX, USA) instructions (n = 3). The TER was calculated through the formula: TER (Ω cm^2^) = [treatment resistance (Ω) − background resistance (Ω)] × membrane area (cm^2^).

### 4.13. Immunofluorescence (IF) Staining

IF staining of SCs was conducted according to a previous study [66]. Briefly, SCs were fixed in 4% paraformaldehyde for 30 min, permeabilized with 0.5% Triton X-100 for 15 min, and blocked with 1% BSA for 1 h. Then, SCs were incubated with anti-WT1 (1:50, Cat# ab267377, Abcam, Cambridge, MA, USA), anti-Vimentin (1:100, Cat# ab92547, Abcam, Cambridge, MA, USA), anti-ZO-1 (1:100, Cat# ab276131, Abcam, Cambridge, MA, USA), anti-Occludin (1:100, Cat# ab216327, Abcam, Cambridge, MA, USA), anti-Claudin-11 (1:100, Cat# 36-4500, Thermo Fisher Scientific, Waltham, MA, USA), anti-Connexin-43 (1:100, Cat# ab217676, Abcam, Cambridge, MA, USA), anti-N-cadherin (1:100, Cat# ab207608, Abcam, Cambridge, MA, USA), and anti-LC3B (1:200, Cat# ab192890, Abcam, Cambridge, MA, USA) overnight at 4 °C. Then, the SCs were incubated with secondary antibody IgG H&L (1:1000, Cat# ab150073, Abcam, Cambridge, MA, USA) at room temperature for 1 h, followed by the nuclei staining of DAPI (Cat# ab104139, Abcam, Cambridge, MA, USA) for 10 min. The images were captured by a fluorescence microscope (Olympus BX53, Tokyo, Japan).

### 4.14. Hoechst 33342 Staining

SCs were fixed in 4% formaldehyde for 30 min at room temperature, and subsequently, the fixed cells were stained with the Hoechst 33342 solution (Cat# R37605, Invitrogen, Carlsbad, CA, USA) for 30 min. The cells were observed under a fluorescence microscope and photographed (Olympus BX53, Tokyo, Japan).

### 4.15. Flow Cytometry

The Annexin V-FITC/PI apoptosis detection kit (Cat# A10788, Invitrogen, Carlsbad, CA, USA) combined with flow cytometry was adopted for apoptosis detection following the manufacturer’s instruction. Briefly, SCs were suspended with binding buffer, followed by Annexin V-FITC and PI staining in the dark for 15 min at room temperature. The stained cells were analyzed by a flow cytometer (BD FACSAria III, San Jose, CA, USA).

### 4.16. Fluorescent Labeling of Mitochondrial and ER

According to the manufacturer’s instructions, MitoTracker Red (Cat# M22425, Invitrogen, Carlsbad, CA, USA) and ER-Tracker Blue (Cat# E12353, Invitrogen, Carlsbad, CA, USA) were used to observe the morphological changes of mitochondrial and ER, respectively. In brief, SCs were stained with MitoTracker Red or ER-Tracker Blue for 30 min at 37 °C, and the images were captured under a fluorescence microscope (Olympus BX53, Tokyo, Japan).

### 4.17. Statistical Analysis

Data were expressed as the mean ± SEM from at least three independent experiments. SPSS version 20 software (IBM, Armonk, NY, USA) was used for the statistical analyses. One-way analysis of variance (ANOVA) was used to compare the differences among multiple groups, and an independent samples *t*-test was used to compare the differences between two experimental groups. A *p*-value < 0.05 was considered statistically significant.

## 5. Conclusions

**Figure 7:**
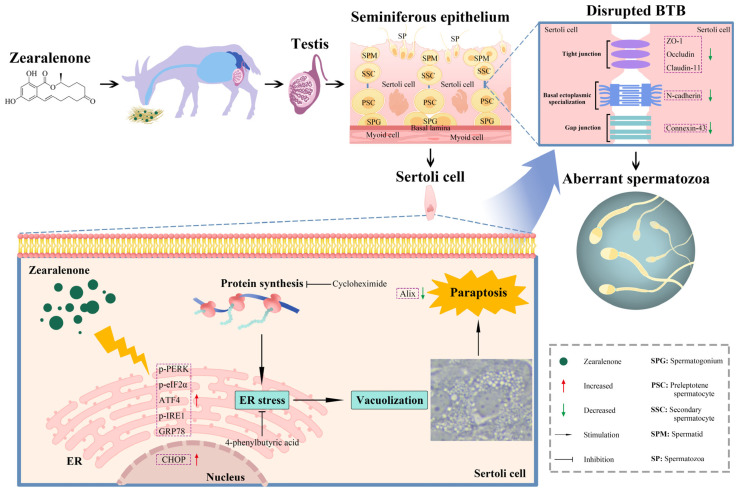
Potential mechanism of ER stress-mediated SC paraptosis regulating ZEA-induced BTB damage in goats.

In this study, ZEA exposure, in vivo, seriously disrupted the testicular structure and BTB integrity and caused the formation of extensive cytoplasmic vacuoles with highly dilative ER in SCs, which preliminarily indicated the occurrence of paraptosis in ZEA-treated goat testes. Further analyses of SCs in vitro revealed that the ZEA treatment led to BTB damage and caused ER-related vacuoles. Importantly, ZEA-induced destruction was significantly alleviated by the CHX-mediated inhibition of paraptosis, which indicated that paraptosis contributed to ZEA-induced SC death. Meanwhile, the expression of ER stress marker proteins was increased after the ZEA treatment but exhibited downregulation under the CHX-mediated inhibition of paraptosis. The ZEA-induced vacuole formation and SC death were significantly blocked by ER stress inhibition under 4-phenylbutyric acid treatment. Taken together, these results revealed that ZEA-induced BTB damage was attributed to the ER stress-mediated paraptosis of SCs in goats (Figure 7).

## Data Availability

Data are contained within the article.
